# Characterizing the vulnerability of frequent emergency department users by applying a conceptual framework: a controlled, cross-sectional study

**DOI:** 10.1186/s12939-015-0277-5

**Published:** 2015-12-09

**Authors:** Patrick Bodenmann, Stéphanie Baggio, Katia Iglesias, Fabrice Althaus, Venetia-Sofia Velonaki, Stephanie Stucki, Corine Ansermet, Sophie Paroz, Lionel Trueb, Olivier Hugli, Judith L. Griffin, Jean-Bernard Daeppen

**Affiliations:** Vulnerable Populations Center, Department of Ambulatory Care and Community Medicine, University of Lausanne & Lausanne University Hospital, Lausanne, CH-1015 Switzerland; Life Course and Inequality Research Center, Faculty of Social and Political Sciences, University of Lausanne, Lausanne, CH-1015 Switzerland; Institute of Social and Preventive Medicine, Lausanne University Hospital, Lausanne, CH-1011 Switzerland; Institute of Higher Education and Research in Healthcare, Lausanne University Hospital, Lausanne, CH-1010 Switzerland; Addiction Switzerland, Research Institute, Lausanne, CH-1001 Switzerland; Department of Community Medicine and Public Health, Lausanne University Hospital, Lausanne, CH-1011 Switzerland; Emergency Department, Lausanne University Hospital, Lausanne, CH-1011 Switzerland; Alcohol Treatment Service, Lausanne University Hospital, Lausanne, CH-1011 Switzerland

**Keywords:** Conceptual framework of vulnerability, Frequent emergency department users

## Abstract

**Background:**

Frequent emergency department (ED) users meet several of the criteria of vulnerability, but this needs to be further examined taking into consideration all vulnerability’s different dimensions. This study aimed to characterize frequent ED users and to define risk factors of frequent ED use within a universal health care coverage system, applying a conceptual framework of vulnerability.

**Methods:**

A controlled, cross-sectional study comparing frequent ED users to a control group of non-frequent users was conducted at the Lausanne University Hospital, Switzerland. Frequent users were defined as patients with five or more visits to the ED in the previous 12 months. The two groups were compared using validated scales for each one of the five dimensions of an innovative conceptual framework: socio-demographic characteristics; somatic, mental, and risk-behavior indicators; and use of health care services. Independent t-tests, Wilcoxon rank-sum tests, Pearson’s Chi-squared test and Fisher’s exact test were used for the comparison. To examine the -related to vulnerability- risk factors for being a frequent ED user, univariate and multivariate logistic regression models were used.

**Results:**

We compared 226 frequent users and 173 controls. Frequent users had more vulnerabilities in all five dimensions of the conceptual framework. They were younger, and more often immigrants from low/middle-income countries or unemployed, had more somatic and psychiatric comorbidities, were more often tobacco users, and had more primary care physician (PCP) visits. The most significant frequent ED use risk factors were a history of more than three hospital admissions in the previous 12 months (adj OR:23.2, 95%CI = 9.1-59.2), the absence of a PCP (adj OR:8.4, 95%CI = 2.1-32.7), living less than 5 km from an ED (adj OR:4.4, 95%CI = 2.1-9.0), and household income lower than USD 2,800/month (adj OR:4.3, 95%CI = 2.0-9.2).

**Conclusions:**

Frequent ED users within a universal health coverage system form a highly vulnerable population, when taking into account all five dimensions of a conceptual framework of vulnerability. The predictive factors identified could be useful in the early detection of future frequent users, in order to address their specific needs and decrease vulnerability, a key priority for health care policy makers. Application of the conceptual framework in future research is warranted.

**Electronic supplementary material:**

The online version of this article (doi:10.1186/s12939-015-0277-5) contains supplementary material, which is available to authorized users.

## Background

A heterogeneous subgroup of patients uses the emergency department (ED) frequently and accounts for a substantial proportion of all ED visits in developed countries [1]. These “frequent users” have a high burden of complex social and medical needs and can be considered a vulnerable population [2]. Our study is in response to the calls of several authors [3–5] to identify the needs of those at risk of future frequent ED use, by examining the predictive risk factors of frequent ED use and by applying a robust and innovative conceptual framework of vulnerability to characterize this population.

Characterizing vulnerable persons or populations is challenging [6]. Vulnerability has been defined as “an identifiably increased likelihood of incurring additional or greater wrong” [6]. Based on the World Health Organization’s dimensions of health (physical, psychological, and social) [7], Aday [8] introduced a framework for studying vulnerable populations, which includes these three dimensions, with the addition of access to, and cost and quality of, care as variables. Recent studies evaluating the medical and social factors associated with frequent ED use have demonstrated the compounding effect of these variables taken together [2, 9]. Based on these results, the Lausanne University Hospital created a clinical evaluation grid of vulnerability. This tool was used to assess the multiple dimensions of vulnerability among ED patients, with a particular focus on frequent users [10]. Based on the clinical experience of using this evaluation grid over 5 years, we created a research-oriented, conceptual framework of vulnerability involving five dimensions: 1) socio-cultural and demographic background (social determinants of health, deprivation, origin, and nationality), 2) at-risk behaviors (addictions to alcohol, tobacco, and/or drugs; violence and/or sexual risk behaviors), 3) somatic health (diagnosis, comorbidity and subjective health), 4) mental health (diagnosis, comorbidity, and subjective health), and 5) health care consumption (resources involved in the previous 12 months).

### The case of frequent ED users

Frequent ED users, by definition, are patients who visit ED on multiple occasions. Therefore, they already meet one criterion of vulnerability from our conceptual framework–namely, health care consumption. Indeed, frequent ED users tend to have higher hospital admission rates [3, 11–14], and have higher or lower triage scale levels, depending on the definition of frequent ED use [15, 16]. They also use non-ED based health care services, such as primary care physicians (PCPs), social workers, and psychiatrists, more frequently than non-frequent users [4, 12]. Frequent ED users have been described in several countries recently [14, 17, 18] including Switzerland [2, 9].

A picture of frequent ED users is emerging from the literature demonstrating the presence of many dimensions of vulnerability beyond high health care consumption. Compared to non-frequent ED users, they have higher rates of social isolation [4], lower socio-economic status [2], a higher burden of psychiatric and medical disease [19], and increased rates of substance abuse [2, 20]. Yet, these findings have not been systematically investigated within a universal health care coverage system, taking into account all of the dimensions of vulnerability.

### The context of the Swiss health system

ED use is partially determined by the characteristics of health care delivery and insurance. In Switzerland, mandatory health insurance is a central feature of the health care system and provides coverage for the full spectrum of health needs, from ambulatory to hospital care [21]. It is estimated that more than 99 % of the population in Switzerland is insured [22]. Insurance premiums are paid by individuals independently of earnings, except for subsidies provided to citizens with low incomes [23, 24]. Deductibles range from CHF 300 to 2500 (279 to 2,325 USD in 2009), with a maximum of CHF 700 (651 USD in 2009) out-of-pocket expenditures charged after the deductible is reached. Cost for outpatient and ED visits are similar and determined by a standardized fee for service scale [25]. Two recent studies conducted in the French-speaking part of Switzerland report that between 10.7-13.8 % of insured patients did not seek out health care during the previous year for economic reasons [26, 27]. Given high out-of-pocket expenditures may be a barrier to accessing appropriate health care in Switzerland [28], avoiding preventive or elective health care for economic reasons could be one of the factors that motivate those who are less well-off and in poorer health to use the ED more frequently than less vulnerable patients.

In this system, patients are free to choose any practitioner [29]. Both ED and PCP may be accessed relatively expediently. It is estimated 93 % of patients in Switzerland are able to make a same-or next-day appointment with a doctor or nurse, while only 2 % reported waiting six or more days to see a provider. In terms of emergency care, 44 % reported waiting less than 30 min to be treated, and only 6 % waited four or more hours [30]. In 2013 49 % of Swiss participants in an international survey reported it was very/somewhat easy getting care after hours [31].

### The aim

The aim of this study was to characterize frequent ED users and to define the risk factors of frequent ED use, within a universal health care coverage system, applying a robust conceptual framework of vulnerability.

## Methods

### Study design, setting, and ethics statement

We used a controlled, cross-sectional design to compare frequent and non-frequent users of the ED at the Lausanne University Hospital, in Switzerland. Using validated scales, we applied the five dimensions of the conceptual framework of vulnerability. This design improves the reproducibility of observations, and strengthens evidence. The ED serves as both a first-response facility for the Lausanne area, and an entry point to the tertiary care facility for the canton (or *State*) of Vaud (catchment area of 770,000 people). The ED has 35,000 visits annually. During the daytime, patients with low triage scores are diverted to an urgent care clinic, which is not part of the current study (15,000 visits annually). The study took place from October 2009 to June 2010. Participation in the study consisted of an interview during which a questionnaire was completed, partially self-administered and partially administered by the research team. All reporting is based on the Strengthening the Reporting of Observational Studies in Epidemiology (STROBE) statement [32].

### Study participants

In line with other recent studies, frequent ED users were defined as patients who had visited the ED at least five times in the previous 12 months, including the index visit during the study period [13, 14]. All patients falling into the frequent user category were automatically identified through a computerized alert system, and were invited to participate in the study. Patients with one to four ED visits in the previous 12 months served as the control group of non-frequent users. The control group was selected as follows: 1) at the beginning of the inclusion phase, one non-frequent ED user for four frequent ED users (the tenth to arrive after admission of the last frequent user); 2) later, one non-frequent ED user for each frequent ED user (the tenth to arrive after admission of the last frequent user); and 3) at the end of the inclusion phase, one non-frequent ED user per day (selection by computerized random number generation). This procedure was applied to address the fact that a decreasing number of frequent ED users were being included in the study.

Patients visiting the ED were eligible for study inclusion if they: 1) were at least 18 years old, 2) did not have any severe cognitive impairment, and 3) were not triaged to specialized EDs (psychiatric, gynecologic, or pediatric). To help offset any travel expenses, reimbursement vouchers for 30 Swiss Francs (USD 28 (in 2009)) were given to patients returning to the hospital solely for the interview. An interpreter was available to those unable to speak or understand French. Patients were excluded from all analyses if they did not complete at least 70 % of the questionnaire.

### Sample

During the study period, 24,277 patients attended the ED, 351 of whom met the definition of frequent user (1.4 % of all ED patients). From the frequent users identified, 46 were excluded (severe cognitive impairments *n* = 31, death *n* = 8, age <18 *n* = 5, no translator *n* = 2) and 305 were invited to participate in the study. Frequent users, as a group, made 2,030 visits in the year prior to enrollment.

A total of 226 frequent ED users completed the questionnaire (i.e., 74 % of frequent ED users invited to participate in the study; 64 % of all frequent ED users). Frequent ED users interviewed did not differ from those who were not interviewed (79 of 305) in regards to gender, age, month and day of attendance, or mean number of attendances in the previous 12 months. A total of 173 non-frequent ED users were included in the control group. Compared to all ED users (*n* = 27,799) in 2009, the control group was similar in terms of case severity and time of day presenting to the ED, but young men were under-represented.

### Data collection

The study team included three research assistants: two nurses and one psychologist. Individuals from the team approached eligible patients in the triage area, waiting room, ED examination room, or hospital room after presentation to the ED. After obtaining written informed consent from the patient, a research assistant administered the main questionnaire, except for the sexual risk behavior items, which were self-administered for privacy reasons (Additional file 1). The self-administered part of the instrument was translated into German, English, Spanish, Portuguese, Serbian, Albanian, Turkish, Tamil, Arabic, and Somali, the ten most common languages spoken by non-French speaking patients of the Lausanne University Hospital, and back translated into French to check the validity of the translation. The interviews took 45 min on average, ranging from 20 to 140 min. For participants unable to give informed consent or to be interviewed in person during their ED stay (e.g., intoxicated patients or weekend patients), phone or home interviews were conducted.

### Outcome measurements

Information was collected on the different dimensions of vulnerability using validated scales (Table 1), applying the conceptual framework described earlier.

**Table 1:** Conceptual vulnerability framework for frequent emergency department users: measurement instruments for social characteristics; somatic, mental, and risk behavior indicators; and health care use

Dimension	Indicator	Measurement^a^
Social-cultural and demographic	Age (years)	Years
	Gender	Gender
	Country of birth	0 = Low/middle-income country; 1 = high-income country
	Education	0 = None, incomplete, primary; 1 = Secondary; 2 = Tertiary
	Marital status	0 = Single; 1 = Married/civil partnership; 2 = Separated/divorced; 3 = Widowed
	Household income (per month)	1 = < CHF 3′000 vs 0 = CHF 3′000 or more [3′000–5′999/more than 6′000/no information]
	Income source	1 = welfare (unemployed) vs 0 = regular income (employed)
	Violence (past 12 months)	PVS: 1 = experienced violence vs 0 = no violence [33]
	Social support	MSSS-5 [34]
	Social position	Self-rated, 10-level, visual, non-numerical scale from 1 = underprivileged to 10 = privileged [35]
	French proficiency	1 = less than good vs 0 = good
	Distance of home from ED	1 < 5 km vs 0 = 5 km or more
Somatic	ED primary diagnosis	MDC classification
	Severity	1 = urgent vs 0 = non-urgent
	Comorbidity	Charlson-Age comorbidity index [36, 37]
	Chronic disease	1 = yes vs 0 = no, based on MDC classification
	Subjective well-being	SF-12v2: physical component [38]
Mental	Comorbidity	Mood, anxiety, or panic disorder (Prime MD: 1 = 1-3 disorders vs 0 = no disorder [39]), M.I.N.I (1 = PTSD vs. 0 = no PTSD [40]); 1 = comorbidities vs 0 = no comorbidity
	Chronic disease	1 = yes vs 0 = no, based on MDC classification
	Subjective well-being	SF-12v2: mental component [38]
Risk behavior	Alcohol consumption	ASSIST: 1 = moderate-high risk vs 0 = no–low risk [41, 42]
	Lifetime illicit drug use	ASSIST: 1 = illicit drug use vs 0 = no use [41, 42]
	Tobacco consumption	HSI: 1 = medium or high score (1–6) vs 0 = low score (0–1) [41–43]
	Sexual risk behaviors	Separate self-administered questionnaire^b^ [44], 1 = sexual behavior at risk; 0 = no at risk sexual behavior
Health care use	PCP visits	0 = no PCP; 1 = PCP, but no visit; 2 = 1 or more PCP visit(s) [1–2 visit(s)/3–6 visits/more than 6 visits]
	Specialist visits	0 = 0 visits; 1 = 1-2 visits; 2 = 3 or more visits
	Hospital admissions	0 = 0 stays; 1 = 1-2 stays, 2 = 3 or more stays
	Use of other EDs	1 = yes vs 0 = no

^a^in […], codes used in primary analysis: due to non-significant results, some categories have been collapsed

^b^See Supplement 1 for a full English version of the questionnaire

*ASSIST* alcohol, smoking, and substance involvement screening test, *CHF* Swiss Francs (in January 2009, 1 Swiss Franc = USD 0.9); *ED* emergency department, *HSI* heaviness of smoking index, *km* kilometer(s), *MDC* major diagnostic categories, *M.I.N.I.* mini-international neuropsychiatric interview, *MSSS-5* modified social support survey, *PCP* primary care practitioner, *Prime MD* primary care evaluation of mental disorders, *PVS* partner violence screen, *SF-12v2* 12-item short-form health survey, version 2

Most data were obtained directly from patients (by self-report on questionnaires). The frequency of ED use was collected from ED administrative databases, and the severity of cases at presentation and primary ED diagnoses were abstracted from the medical chart pertaining to current ED visit at the time of enrollment.

### Statistical analysis

Descriptive statistics were computed using means and standard deviations (or median and inter-quartile range, depending on the distribution) for continuous variables, and frequencies and percentages for categorical variables. Frequent and non-frequent ED users were compared using independent t-tests or Wilcoxon rank-sum tests for continuous variables, and Pearson’s Chi-squared test or Fisher’s exact test for categorical variables.

To identify the risk factors of frequent ED use, univariate and multivariate logistic regression models were used to analyze the impact of social-cultural and demographic characteristics, somatic and mental health conditions, at- risk behaviors, and health care use variables. A stepwise selection procedure was used [45]. Odds ratio (OR) for univariate analysis and adjusted OR (adj OR) for multivariate analysis, and their associated 95 % confidence interval (CI), were reported for each estimated parameter in the final model. Due to the underrepresentation of young men in the control group, we controlled for gender and age in the analysis. All analyses were performed using Stata (StataCorp. 2009. *Stata Statistical Software: Release 11*. College Station, TX (USA): StataCorp LP) with the threshold for statistical significance set at *p* = 0.05.

### Ethics, consent and permissions

The study received approval from the Ethics Committee of the University of Lausanne (reference number 156/09). We obtained written consent from all study participants.

## Results

### Baseline characteristics of frequent ED users compared to non-frequent ED users

In terms of the socio-cultural and demographic characteristics presented in Table 2, frequent and non-frequent ED users were comparable with regards to gender and education. Frequent ED users were significantly younger than controls (*p* < 0.05) and more likely to have been born in a low- or middle-income country (*p* < 0.001). In the social dimension, a greater proportion of frequent users had a low monthly household income (*p* < 0.001), and frequent users were more likely to be unemployed or dependent on social welfare (*p* < 0.001). Frequent users reported higher rated of suffering violence in the previous 12 months (*p* < 0.001), and rated their social support (*p* < 0.05) and social position (*p* < 0.001) lower than non-frequent users. Finally, they were less proficient in French (*p* < 0.001), and lived closer to the ED (*p* < 0.001) than controls.

**Table 2:** Characteristics of frequent users of the ED compared to non-frequent users (control group), N = 399

Characteristics and indicators	Variables	Frequent users *n* = 226 Mean (SD), n (%), or Median [IQR]	Control group *n* = 173 Mean (SD), n (%), or Median [IQR]	*p*-value^*^
Demographic	Age (years)	51.5 (21.5)	56.2 (22.6)	<0.05
	Gender (female)	102 (45.1)	95 (54.9)	ns
	Country of birth (low/middle-income countries)	58 (25.7)	17 (9.8)	<0.001
	Education			ns
	None, incomplete, primary	68 (30.2)	52 (30.1)	
	Secondary	115 (51.1)	82 (47.4)	
	Tertiary	42 (18.7)	39 (22.5)	
	Marital status			<0.05
	Single	76 (33.6)	48 (27.8)	ns
	Married/civil partnership	67 (29.7)	60 (34.7)	ns
	Separated/divorced	59 (26.1)	32 (18.5)	ns
	Widowed	24 (10.6)	33 (19.1)	<0.1^**^
Social	Household income < CHF 3,000	90 (39.8)	34 (19.7)	<0.001
	Income source (Unemployed or dependent on welfare or social welfare)	105 (46.5)	26 (15.0)	<0.001
	Violence (yes)	57 (25.5)	19 (11.0)	<0.001
	Social support [*n* = 393]	75 [40;100]	80 [58;100]	<0.05
	Social position [*n* = 385]	5 [3–6]	5 [5–7]	<0.001
	French proficiency (less than very good)	79 (35.1)	28 (16.2)	<0.001
	Distance from home to ED <5 km	162 (73.3)	96 (55.5)	<0.001
Somatic	ED primary diagnosis			<0.001
	Circulatory	29 (12.8)	24 (13.9)	ns
	Digestive	24 (10.6)	13 (7.5)	ns
	Infectious diseases	47 (20.8)	28 (16.2)	ns
	Injury	22 (9.7)	42 (24.3)	<0.001^**^
	Substance use & mental disorder	31 (13.7)	7 (4.1)	<0.01^**^
	Other	73 (32.3)	59 (34.1)	ns
	Severity (triaged as urgent)	84 (37.2)	56 (32.4)	ns
	Comorbidity index [*n* = 390]	3 [1;6]	2 [0;5]	<0.01
	Chronic disease (yes)	116 (51.8)	102 (60.0)	ns
	Subjective well-being^a^ [*n* = 383]	38.6 (13.3)	41.9 (13.4)	<0.05
Mental	Comorbidity (yes)	119 (53.9)	45 (26.6)	<0.001
	Chronic disease (yes)	68 (30.4)	21 (12.4)	<0.001
	Subjective well-being^b^ [*n* = 383]	40.9 (14.2)	45.3 (14.1)	<0.01
Risk behavior	Alcohol use (moderate to severe)	54 (24.3)	12 (6.9)	<0.001
	Illegal drug use (yes)	38 (17.0)	13 (7.5)	<0.01
	Tobacco consumption (≠ no use)	67 (29.7)	24 (13.9)	<0.001
	Sexual behaviors (yes)	32 (19.8)	14 (10.5)	<.05
Health care use	PCP, number of visits			<0.001
	No PCP	50 (22.4)	18 (10.5)	<0.01^**^
	PCP, no visit	15 (6.7)	18 (10.5)	ns
	PCP, 1 or 2 visits	26 (11.7)	51 (29.7)	<0.001^**^
	PCP, 3 to 6 visits	54 (24.2)	50 (29.1)	ns
	PCP, 7 or more visits	78 (35.0)	35 (20.4)	<0.01^**^
	Medical specialist visits			<0.001
	No visit	73 (32.3)	81 (47.4)	<0.01^**^
	1 or 2 visits	35 (15.5)	48 (28.1)	<0.01^**^
	3 or more visits	118 (52.2)	42 (24.6)	<0.001^**^
	Hospital admissions			<0.001
	No admissions	50 (22.4)	99 (57.9)	<0.001^**^
	1 or 2	50 (22.4)	62 (36.3)	<0.01^**^
	3 or more	123 (55.2)	10 (5.9)	<0.001^**^
	Use of other EDs (yes)	60 (26.6)	32 (18.5)	<0.001^**^

^*^ *ns* non-significant

^**^A Bonferroni correction for post hoc testing was carried out on this *p*-value (*p*-value multiplied by the number of modalities)

Independent *t*-tests or Wilcoxon rank-sum tests for continuous variables and Pearson Chi-squared tests for categorical variables were used. Twenty-seven variables were tested; the results are presented without correction for multiple testing on these variables. When correcting for multiple tests, all *p*-values lower than 0.0018 are still significant

*CHF* Swiss Francs (in January 2009, 1 Swiss Franc = USD 0.9), *ED* emergency department, *PCP* primary care practitioner

^a^Short Form Health Survey (SF-12) [35], physical component summary

^b^Short Form Health Survey (SF-12) [35], mental component summary

Concerning somatic vulnerability, frequent ED users had more primary diagnoses (*p* < 0.001), a higher comorbidity index (*p* < 0.01), and less subjective well-being related to their somatic health (*p* < 0.05) compared with non-frequent users.

In the mental health vulnerability dimension, frequent ED users were more likely to have mental comorbidities (*p* < 0.001), chronic mental illness (*p* < 0.001), and lower levels of subjective well-being related to their mental health (*p* < 0.01) than controls.

With regard to at-risk behaviors, frequent ED users had higher rates of moderate-to-severe alcohol use (*p* < 0.001), illicit drug use (*p* < 0.01), and tobacco use (*p* < 0.001) compared to non-frequent ED users. They also reported more risky sexual behavior (*p* < 0.05).

Finally, within the health care use dimension, the median annual number of ED visits was five for frequent ED users compared to one for controls. All patients in the study had health insurance. Frequent ED users were less likely to have a PCP than controls (*p* < 0.01); however, if they did have a PCP, they were more likely to visit the provider more than seven times per year (*p* < 0.01). Frequent ED users were also more likely to visit medical specialists and to have been hospitalized in the previous 12 months (*p* < 0.001).

Of note, when patients disclosed certain risk factors, including a history of violence or substance abuse, they were referred to the hospital’s case management team or to specialized departments (such as the Violence Medical Unit), in order to receive appropriate care (contingent on the patient’s consent).

### Risk factors for frequent ED use

After adjusting for gender and age, 12 variables were found to be significantly associated with frequent ED use. Table 3 displays the results of the final multivariate logistic regression model.

**Table 3:** Risk factors associated with frequent ED use, *N* = 364

	Risk factors	Unadjusted OR	Adj. OR (95 % CI)	*p*-value^*^
Demographic	Age (by 10 years over 18 years)	0.9 (0.8-1.0)	0.8 (0.6-1.0)	<0.05
	Gender (female)	0.7 (0.5-1.0)	0.4 (0.2-0.9)	<0.05
Social	Household income < CHF 3′000	2.7 (1.7-4.3)	4.3 (2.0-9.2)	<0.001
	Distance from home to ED <5 km	2.2 (1.4-3.4)	4.4 (2.1-9.0)	<0.001
Somatic	ED primary diagnosis (ref. = circulatory system)	1.0	1.0	
	Injuries	0.4 (0.2-0.9)	0.3 (0.1-0.9)	<0.05
	Other	1.4 (0.7-2.4)	0.9 (0.3-2.6)	ns
	Comorbidity index	1.1 (1.0-1.2)	1.2 (1.1-1.4)	<0.01
	Chronic disease (yes)	0.7 (0.5-1.1)	0.3 (0.1-0.7)	<0.01
Mental	Comorbidities (yes)	3.2 (2.1-4.9)	2.3 (1.2-4.7)	<0.05
Risk behavior	Tobacco consumption (≠ no use)	2.6 (1.6-4.4)	2.6 (1.1-5.9)	<0.05
Health care use	PCP and number of visits (ref. = PCP, no visits)	1.0	1.0	
	No PCP	3.3 (1.4-8.0)	8.4 (2.1-32.7)	<0.01
	PCP, 1 or more visits	1.4 (0.7-2.9)	1.0 (0.3-3.0)	ns
	Hospital admissions (ref = 1 or 2)	1.0	1.0	
	0 admissions	0.6 (0.4-1.0)	0.3 (0.1-0.5)	<0.001
	3 or more admissions	15.3 (7.2-32.1)	23.2 (9.1-59.2)	<0.001
	Use of other EDs (yes)	1.6 (1.0-2.6)	2.7 (1.2-6.1)	<0.05

^*^ *ns* non-significant

Sensitivity = 85.8 %, specificity = 81.3 %, and area under curve (AUC) = 0.921

*CHF* Swiss Francs (in January 2009, 1 Swiss Franc = USD 0.9), *ED* emergency department, *PCP* primary care physician

The risk of being a frequent user was higher for patients with a low monthly household income compared to those with higher incomes or missing income information (adj OR = 4.3; 95 % CI = 2.0-9.2); for patients living close to the ED versus those living further away (adj OR = 4.4; 95 % CI = 2.1-9.0); for a somatic (adj OR = 1.2; 95 % CI = 1.1-1.4) or a mental (adj OR = 2.3; 95 % CI = 1.2-4.7) comorbidity change of one unit according to the Charlson comorbidity index; for those with moderate to severe tobacco use compared to lower-level users or nonsmokers (adj OR = 2.6; 95 % CI = 1.1-5.9); for patients who had no PCP compared to those who had (adj OR = 8.4; 95 % CI = 2.1-32.7); for patients with three or more admissions to the hospital versus those with 1–2 admissions in the previous 12 months (adj OR = 23.2; 95 % CI = 9.1-59.2); and for patients that attended other EDs versus those who did not (adj OR = 2.7; 95 % CI = 1.2-6.1).

The risk of being a frequent user was lower for women (adj OR = 0.4; 95 % CI = 0.2-0.9); for patients attending for injuries versus those attending for circulatory system ailments (reference category) (adj OR = 0.3; 95 % CI = 0.1-0.9); for those with chronic somatic conditions compared to those without them (adj OR = 0.3; 95 % CI = 0.1-0.7); and for those with no hospital admissions compared to those with one or two in the previous 12 months (adj OR = 0.3; 95 % CI = 01-0.5).

## Discussion

The aim of the present study was to examine whether frequent ED users could be characterized as a vulnerable population and to define the risk factors of frequent ED use, through a novel conceptual framework of vulnerability. Our study shows that frequent ED users should be considered a highly vulnerable population, given that they were found to have significantly more vulnerability factors than non-frequent users across all five dimensions of the conceptual framework. Additionally, specific vulnerability factors were found to be predictive factors of frequent ED use, which supports the adoption of the vulnerability framework as a tool in clinical practice and future research.

The majority of the results from the univariate analysis are consistent with findings previously described in the literature. One notable exception was that frequent ED users were found to be significantly younger than the control group. In other studies they are either found to be older [13, 18, 46] or no association between age and frequent ED use is found [2, 47]. This finding could be related to the fact that younger men were under-represented in the control group. Frequent ED users in our study were more likely to have been born in a low- or middle-income country and to be less proficient in the local language (French). A quarter of the frequent users in the study were migrants from low- and middle-income countries, which far exceeds the proportion found in the general population of Switzerland during that time [48]. Several factors across all of the vulnerability dimensions are consistent with findings from previous studies, including:

- Social factors: lack of employment [2, 47], dependence on social welfare, [2] low income [11, 46, 49], limited social support [19] and living close to the ED [2, 18, 47].
- Somatic factors: higher Charlson comorbidity index, lower subjective well-being [1].
- Mental factors: chronic mental disease, mental comorbidities, and lower levels of subjective well-being related to their mental health [13, 19, 50].
- Risk Behaviors: tobacco, alcohol, illicit drug use, and risky sexual [47].
- Health care use: hospital admission [3, 14], visiting more than one ED in the previous 12 months, and more visits to medical specialists [4, 12].

The predictive factors for being a frequent ED user identified in the multivariate analysis also yielded several expected results which are consistent with prior literature including male sex [16, 18], low income [5], somatic and mental comorbidities [2], tobacco use [47], close proximity to the ED [2] and high overall utilization of health care [1]. Several notable differences were identified, however. Interestingly, the risk of being a frequent user was lower for participants who reported having chronic somatic diseases, in contrast to previous studies in which chronic medical conditions have been associated with frequent ED use [11, 46]. This could be due to the existence of a well-established network for specific diseases (e.g. diabetes or congestive heart failure), which directs patients toward specialized services [51]. Contrary to what is indicated in the literature [47], alcohol use was not an independent risk factor for frequent ED use in our model, probably because all patients who presented with moderate to severe alcohol use also had mental comorbidities and were attended to by the psychiatrists in our ED setting. Finally, having no PCP was more common for frequent users in our study, in contrast to findings in the literature: some authors [11, 19] report that frequent users are more likely to have a PCP, while in other studies [2, 47] no association is found. Lucas and Sanford [3] show, in an uncontrolled design, that frequent users have access to other sources of primary care, and Hunt et al. [49] report that having a regular source of care is associated with frequent ED use.

Frequent ED users in this controlled, cross-sectional study were found to be a vulnerable population across all the dimensions of our framework, despite the fact that all these individuals were insured and that they were managed in a system providing high-quality health care [21, 29]. This finding is in accordance with various studies where medical and social vulnerability factors have been found to be associated with frequent ED use, independent of health care coverage [2, 4, 12, 19, 50]. In the current debate regarding the need to expand insurance coverage in order to improve health care access [52, 53], we have observed that frequent ED users still exist in universal health coverage settings. As in our case; they are a vulnerable population and their profiles are similar to those observed in other health care systems. Consequently, in order to address the issue of frequent ED use, health systems with or without universal coverage must proactively identify and anticipate the needs of frequent ED users.

There are several limitations to this study. First, patients presenting during the daytime with low severity scores were directed to an urgent care clinic after triage by ED nurses, and thus were not included. None of the visits on this pathway were included when frequency of use was calculated. This triage pathway was in place because, at the time of the study, the Lausanne University Hospital ED was the referral center during the 2009 influenza A (H1N1) pandemic [54]. As a result, there was overcrowding in the ED. This unexpected set of circumstances caused significant logistical problems for our study. It is possible that the patients we are describing may be a population of more severe cases, although patients with low triage scores presenting at night (when the urgent care clinic was closed) were included. Secondly, although we based our case definition on a careful search and interpretation of the literature [13, 14], the same definition is not universally applied in the literature, as previously discussed. Thirdly, data on health care use were provided by patients’ self-reports and can be subject to bias, since respondents commonly underreport their use of health services [4]. Therefore, our findings may underestimate the extent of health service use among all patients. Finally, our findings are based on a study conducted at a single, urban ED at a large, public teaching hospital; the external validity for other types of ED settings in Switzerland has not been established. Despite these limitations, the use of a clinically-rooted conceptual framework of vulnerability, which is based on international literature regarding vulnerability, [6–8], as well as our own research [2, 9] and clinical experience [10], ensures that all known factors related to vulnerability have been taken into consideration. Additionally, the controlled design is a noted strength, since frequent ED users are compared to non-frequent users within the same facility.

## Conclusions

This study—applying a robust, innovative, and clinically-rooted conceptual framework of vulnerability—showed that frequent users of an ED within a universal health care coverage system represent a socially and medically vulnerable population, cumulating different dimensions of vulnerability. The risk factors found in our study could be used in clinical practice to identify those at risk for future frequent ED use. By doing so, we may be able to develop more personalized and effective interventions, and to reduce related disparities and inequities associated with frequent ED use. Finally, the results of this study support the application of this conceptual framework in future research settings and health care policy efforts targeting various vulnerable patient populations, including frequent ED users, so-called “super-utilizers” of other health care services, or other vulnerable populations.

## Additional file

Additional file 1:
**Questionnaire on sexual risk behaviors (self-administered).** (DOC 22 kb)

## Abbreviations

adj OR: adjusted odds ratio
ED: emergency department
PCP: primary care physician
