# Supplementary material for: Characterizing the vulnerability of frequent emergency department users by applying a conceptual framework: a controlled, cross-sectional study
Source: Int J Equity Health. 2015 Dec 9;14:146. doi: 10.1186/s12939-015-0277-5 (PMC4673736; doi:10.1186/s12939-015-0277-5)
Supplement: Additional file 1: — Questionnaire on sexual risk behaviors (self-administered). (DOC 22 kb) [file 12939_2015_277_MOESM1_ESM.doc]

**S1. Questionnaire on sexual risk behaviors (self-administered).**

#### 1. What was your relation with your partner?

Stable/Occasional/Paid or paying

#### 2. Since you have been sexually active, have you ever paid or offered a gift in return for sexual intercourse?

Yes/No

#### 3. Have you ever accepted payment or gifts in return for sexual intercourse?

#### Yes/No

4. During the last 12 months, did anybody force you to have sexual intercourse against your will?

Yes/No

At risk for sexual behavior: answered “Paid or paying” to question 1, or at least one “Yes” to questions 2, 3, and 4.
